# Healthcare Workers’ Perspectives on the E-MOTIVE Postpartum Haemorrhage Bundle in Tanzania: A Qualitative Study of Implementation Facilitators and Challenges

**DOI:** 10.2147/IJWH.S596675

**Published:** 2026-05-06

**Authors:** Fadhlun M Alwy Al‑beity, Amani I Kikula, Akwinata Patrick Banda, Juma Maganya Makungu, Beatrice Erastus Mwilike, Masumbuko Masuke Sambusa, Ard Bernhard Mwampashi, Emmy Metta

**Affiliations:** 1 Department of Obstetrics/Gynaecology, School of Clinical Medicine, Muhimbili University of Health and Allied Sciences, Dar Es Salaam, Tanzania; 2 Department of Clinical Nursing, School of Nursing, Muhimbili University of Health and Allied Sciences, Dar Es Salaam, Tanzania; 3 Department of Behavioral Sciences, School of Public Health and Social Sciences, Muhimbili University of Health and Allied Sciences, Dar Es Salaam, Tanzania

**Keywords:** E-MOTIVE intervention, postpartum haemorrhage, PPH first response bundle, implementations, consolidated framework for implementation research

## Abstract

**Background:**

The World Health Organization proposed a clinical care bundle as a first response for postpartum haemorrhage (PPH), a leading cause of maternal deaths. A large trial was conducted to evaluate its effectiveness, with Tanzania among the participating countries. We explored the experiences of healthcare workers during the early implementation of this bundle within the broader trial.

**Methods:**

An exploratory qualitative study was conducted to understand the early implementation experiences of the PPH bundle. We held six focus group discussions (FGDs) with healthcare workers, clinical leads designated as champions, and research nurses from all six facilities that implemented the intervention in Tanzania. A d-eductive qualitative thematic analysis was performed using the Consolidated Framework for Implementation Research to structure our findings. We reported on four domains: the intervention and its implementation, individual attributes, and the inner and outer contexts.

**Results:**

Healthcare workers reported positive experiences during implementation of the PPH first-response bundle. The innovation included several known components: calibrated drape for early detection, on-site training, facility audits, champions and the PPH care bundle comprising uterine massage, oxytocics, tranexamic acid and intravenous fluids. Individual participants appreciated the multi-component strategies that addressed chronic issues, including the lack of objective measurement of blood loss, inadequate skills, and supply shortages. Additionally, local clinical leads who served as champions increased local ownership and accountability. Facility-level challenges included staff rotation and a lack of clear communication, particularly when managing at-risk women. The availability of external support for training, supplies, and drugs required to administer the bundle, along with strong support from the districts and regional health management teams, enhanced the adoption and implementation process.

**Conclusion:**

The components of the bundle and the implementation strategies were well received and perceived as reducing the risk of severe postpartum haemorrhage and potentially saving lives. The perceived successes were contributed by the interconnectedness of the different strategies used. Facilities and countries aiming to scale need to harness these strategies that target the health system. In addition, there is a need to strengthen local facility audits, document and address chronic challenges affecting staff dynamics and supplies. District, regional and national leadership in adopting and integrating new clinical practices are key.

## Background

Postpartum haemorrhage (PPH) complicates 6–10% of all vaginal deliveries.^1^ It is responsible for nearly one-quarter of maternal deaths worldwide.^1^ PPH is defined as excessive bleeding after childbirth of ≥ 500 mL, primarily occurring within the first 24 hours of childbirth. The consequences of PPH include increased need for resource-intensive interventions like blood transfusion, intensive care unit admission, prolonged hospital stay, postpartum anaemia and organ dysfunction.^2^ The social, economic, and health system consequences of PPH are beyond the individual woman and her family, leading to increased health system costs.^2–5^

Risk identification, Active Management of the Third Stage of Labour in all women, and early detection and management of PPH are key to reducing PPH.^6–9^ The majority of women who develop PPH have no known risk factors, leaving prevention and early management to avert progression on the vigilance, knowledge and skills of the healthcare worker available at the bedside. This requires competent healthcare workers and an enabling system; adequate medical supplies and drugs; blood and blood products; a functioning referral system; and access to senior, skilled personnel when required.^10^

The World Health Organization (WHO) and partners such as the International Confederation of Midwives (ICM) and the Federation Internationale de Gynécologie et d’Obstétrique (FIGO) have provided guidelines on the prevention and management of PPH; however, these guidelines are often not implemented at the bedside, especially in low and middle-income countries (LMIC).^8^ Lack of provider knowledge and skills, under-resourced and constrained health systems are among the challenges contributing to low guideline implementation.

In 2020, the WHO recommended a first-response bundle for PPH.^11^ A “clinical-care bundles” concept is defined as a “*small set [3–5] of evidence-based interventions implemented together for a specific condition*.^11^ *Such bundling has led to significantly better adherence to evidence-based practices and better* outcomes.^12^ The components of the WHO PPH first response bundle were: Early detection using a calibrated drape, uterine Massage, administration of additional Oxytocic drugs, tranexamic acid, intravenous fluids, and escalation (if all were ineffective), acronymed as E-MOTIVE. The trial had a formative phase that explored healthcare workers’ understanding of PPH, knowledge, skills, motivation and barriers that hinder effective and timely diagnosis and management of PPH. Identified barriers included disorganized and unavailable essential medical supplies and drugs, lack of knowledge and skills of guidelines, and staff shortages, all leading to non-adherence to treatment guidelines.^13–16^ Findings from the formative work were used to design specific implementation strategies: involvement of local facility champions, a PPH trolley/kit, and monthly facility audit newsletters.^13^,^17^

The effectiveness of the E-MOTIVE bundle was assessed in four high PPH burden countries: Kenya, Nigeria, Tanzania, and South Africa, between 2021 and 2023.^18^ The study reported a statistically significant 60% reduction in severe PPH. Additional evaluations reported implementation of the bundle was feasible, acceptable, well adhered to, and cost-effective.^19^,^20^

Scaling up the implementation of the E-MOTIVE bundle could substantially reduce maternal mortality within and beyond the study countries. The present study aimed to explore the early implementation of the E-MOTIVE intervention in the study sites in Tanzania, to derive knowledge to guide future implementation and potential scale-up in similar contexts.

## Methods

### Theoretical Framework

The Consolidated Framework for Implementation Research (CFIR) describes factors that facilitate or hinder implementation efforts.^21–23^ The CFIR framework underpins the interconnectedness of its five domains: the intervention, context (internal setting and external structures that affect implementation), individuals’ attributes, and the process of implementation, recognizing that each domain affects the process and outcomes.^21^,^23^,^24^

We used the term “innovation” to denote the practice to be implemented (the E-MOTIVE bundle), as the intervention could be mistaken for the implementation activity itself. Consequently, the domain intervention characteristics of this study are denoted as innovation and its delivery strategies. Individual characteristics refer to the attributes of healthcare workers that influence their perceptions and ability to practice innovation, ultimately affecting their success or failure. The framework describes interactions of different constructs to explain the integration and implementation of the intervention in routine practice.

### Study Design

This qualitative study explored the early implementation experiences of the E-MOTIVE bundle intervention among healthcare workers in Tanzania. For the study, early implementation was defined as occurring within 3 months of the intervention start. This period was crucial to ensure intervention is understood, well practiced, and to address misconceptions and inconsistencies.

### Study Context

Tanzania is among the countries with a high burden of maternal and perinatal mortality and morbidities globally. At the start of the E-MOTIVE study, the maternal mortality ratio was approximately 278 per 100,000 livebirths.^25^ A quarter of these deaths were from PPH.^26^ Factors contributing to high PPH morbidities and mortality include high fertility rate, overwhelmed health systems, lack of and unevenly distributed skilled healthcare workers, and non-functional referral systems.^16^ Tanzania has significant health worker shortages, with 7.2 nurse-midwives and 1.1 physicians per 10,000 population.^27^

The current study was conducted at six intervention facilities of the E-MOTIVE trial, located in semi-urban and rural areas of Tanzania. All participating facilities were owned and funded by the government, with some local income generated through cost-sharing. All facilities procured their medical supplies through the local government system, which was challenged by episodic stockouts, inconsistent quantities and timing, and, often, by women and their families having to contribute to the costs of required supplies.

All six participating facilities offered comprehensive obstetric care; two were high-volume health centres, and four were district hospitals. All had functional theatres for emergency obstetric surgeries, could provide blood transfusions, and had a working referral system to higher levels of care. All facilities averaged 200 to 330 deliveries per month, with annual deliveries of 2000 to 4000.

Childbirth services were provided by nurses, nurse-midwives, clinicians and medical doctors.^28^ The study facilities, like other rural facilities, had significant health worker shortages, with only 40% of the required number of health workers available. No medical specialists were available in any of the study facilities.

Generally, between 12 and 25 nurse-midwives are assigned to the maternity ward (delivery, antenatal, postnatal, and post-op wards), including those on leave. Staff work eight-hour shifts, with more staff assigned to the morning shifts. One nurse or nurse-midwife and one doctor are assigned to oversee routine processes in the labour ward. The E-MOTIVE trial added four licensed research nurse-midwives across all delivery units, thereby increasing the number of providers in each facility.

**Figure 1:**
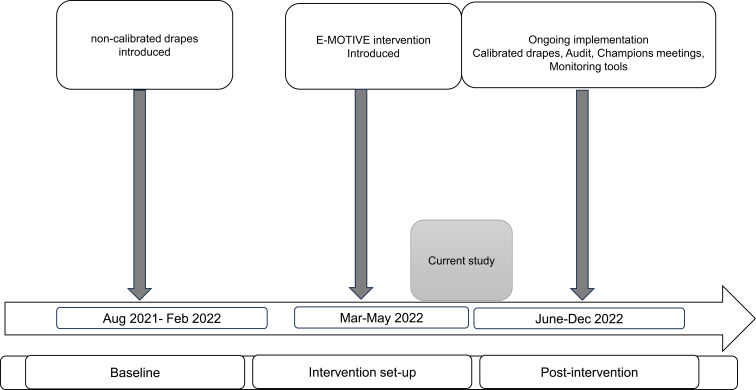
Milestones for the Cluster- randomized E-MOTIVE study and intervention.

Pregnant women in labour are admitted and managed within the delivery unit. Immediately after each normal birth, healthcare workers place a drape to collect all the blood lost. The drape is left in place for 1–2 hours and weighed to measure blood loss. Figure 1 outlines the milestones of the E-MOTIVE trial, within which the current study was done. Details of the main trial are published elsewhere.^18^

### Introducing the E-MOTIVE-Bundle Intervention in Facilities

The intervention began with training facility trainers using the E-MOTIVE training materials developed by Jhpiego, which employed a low-dose, high-frequency training technique, along with Mama Natalie simulators.^29^ These facility trainers, or “E-MOTIVE champions”, accompanied by Jhpiego Master trainers, conducted on-site training for all healthcare workers within the intervention facilities. The initial one-day training was followed by weekly/regular simulation practices. Other implementation strategies introduced in parallel with the on-site training included the PPH trolley, monthly facility audits, calibrated drapes (replacing previously non-calibrated drapes), and champions.

Additional tools were a monitoring checklist with regular patient assessment and documentation of vital signs, such as blood pressure, pulse rate, uterine tone and massage, the amount and rate of blood loss. Further details of the E-MOTIVE project are available elsewhere.^18^

### Study Participants and Sampling

We conveniently recruited health facility implementation teams: E-MOTIVE champions, healthcare workers, and research nurses (Table 1). This sampling method was chosen because the number of eligible providers at each facility was limited, and staff worked on different shifts. Convenience sampling allowed the inclusion of providers who were directly involved in implementing the intervention and were available to participate during the study period.

**Table 1:** Main FGDs of the Local Implementing Teams

FGD Participant Groups	Role
E-MOTIVE Champions	Lead maternity ward healthcare workers, including medical doctors and nurse midwives. They had the authority to initiate, maintain, and sustain the intervention.
Healthcare workers	These were the healthcare workers at the local facilities. They were the primary implementers of the intervention within their setting and had contextual knowledge
Research nurses	These were nurses and midwives employed by the research. They had set precedents for interventions and arrangements, and provided additional support to clinical healthcare workers

### Data Collection Process

In August 2022, three months after the intervention began, the research teams visited various facilities to understand the implementation process and support its implementation. The facilitator and research team comprised an obstetrician/gynaecologist, a social scientist, and midwives; two were male, and two were female. After each visit, they engaged in discussions with the implementation teams. These FGDs were convenient and efficient, fostering authentic interactions and dynamics, as participants already knew each other, thereby reducing social desirability bias. All participants provided their consent for both the discussion and audio recording. There were no refusals to participate in the discussions.

Six focus FGDs were conducted in private rooms within health facilities. Each consists of 6 to 8 participants. One FGD was conducted per health facility. Each FGD included at least two members of each implementor category (Table 1) who were involved in the day-to-day adoption and implementation of the intervention. The FGDs followed a pre-prepared topic guide that was continuously refined as the discussions progressed (Supplementary File 1). All discussions were conducted in the local language, Swahili, were audio-recorded, and lasted between 60 and 120 minutes. Field notes were taken to document nonverbal cues.

The data collection process was iterative, evolving with each FGD, allowing participants to reflect as the implementation continued. Concepts that were challenging and required clarification were explored further in subsequent FGDs. We determined that data saturation was achieved when subsequent FGD discussions yielded no new insights into the CFIR domains and when we had a rich, multifaceted understanding of the key implementation barriers and facilitators.

The focus group discussions were led by an external facilitator who was independent of the trial delivery team. The facilitator used a semi-structured focus group discussion guide to facilitate the discussions. Another team member was present to take detailed notes and handle the audio recordings. Having an outside facilitator helped minimize bias and foster open discussion among participants.

### Data Analysis

All audio-recorded discussions were transcribed verbatim in Swahili Word files within 24 hours of their occurrence. Field notes were expanded and integrated into the transcriptions to provide concise information and enhance clarity. All transcripts were cross-checked against the corresponding audio recordings to verify completeness and accuracy. The transcripts were then translated into English by two bilingual researchers. To ensure accuracy and preserve the original meaning, the English translations were reviewed against the original Swahili transcripts by a bilingual team member, and any discrepancies were discussed and resolved. All translated transcripts were imported into MAXQDA 2022.^30^

Thematic analysis was conducted.^31^ The analysis followed a combined inductive-deductive approach. Initially, the process began with all authors independently, reading and rereading the transcripts to familiarize themselves with the data and generate an initial list of codes directly from the data. All authors then met to discuss and refine the codes, and grouped them into sub-themes. In the next stage, sub-themes were mapped to deductive themes derived from the CIFR framework domains, which were used to organize and present the findings. Therefore, the sub-themes represent concepts that emerged directly from the data, while the main themes correspond to the CFIR framework’s domains. We used the consolidated criteria checklist for qualitative studies to report the findings (Supplementary File 2).

### Trustworthiness

The trustworthiness was ensured through participant validation, transferability, credibility, dependability, and fairness.^32^ The research team summarized and presented key findings to the FGD members to verify, clarify, or add further information. Dependability was ensured through daily reflections and peer review of the entire data collection, management, analysis, and manuscript writing process. The analysis process was iterative to ensure fairness in presenting the findings. All authors participated in the process, from initial coding through analysis and manuscript writing. Discrepancies were resolved through discussions, and attention was given to presenting different opinions as they emerged from the data, even if they represented minority views.

### Reflexivity

The research team was composed of local researchers working within the study facilities to support the main E-MOTIVE trial. They were not part of the training or implementation teams. All members of the research team were experienced qualitative researchers with backgrounds in clinical practice and health systems research. To minimize potential influence on participants’ responses, the focus group discussions were facilitated by an external moderator who was independent of the trial delivery team. A member of the research team attended the sessions to take field notes and help with logistical arrangements.

## Results

A total of 42 participants took part in the FGD discussions: 16 healthcare workers, 12 champions, and 14 research nurses who were directly involved in implementing the intervention at the labour ward/health facility. The median age was 31 years (range 24–44), and the median work experience at the maternity ward was 3 years (range 1–17).

**Figure 2:**
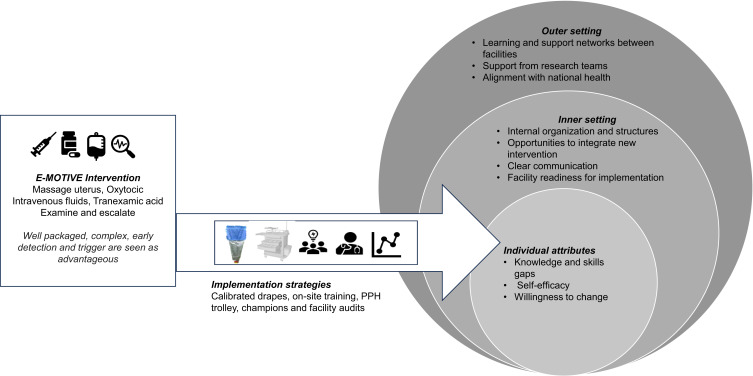
Domains of the CFIR: the E-MOTIVE intervention as an innovation and its implementation strategies, individual attributes, inner facility and organizational structures and outer settings that facilitate or hinder the implementation.

The findings are presented under the four CFIR domains as main themes: intervention characteristics, individual characteristics, inner structure, and outer structure (Figure 2). Each domain has three to six sub-themes (Table 2).

**Table 2:** CFIR Domain, Themes and Sub-Themes

Domain	Theme	Sub-Theme
Innovation	The E-MOTIVE Intervention was well-packaged for implementation.	Evidence: The bundle is based on a combination of well-known and lesser-known interventions.
		Complexity: Several strategies are needed to make it work
		Relative advantage: tools to help with early detection and prompt bundle initiation
Individuals	Individual Characteristics influencing the adoption of intervention into routine practices	Knowledge-skill gap in PPH management practices
		Self-efficacy: healthcare workers felt that all components of the intervention were within their scope of work
		Healthcare workers’ willingness to embrace practice change.
Inner setting	The health facility’s setting facilitates or deters implementation	Internal organization and structures are barriers to the implementation of clinical practices.
		Opportunities to integrate new interventions within the facility culture
		Clear Communication is necessary for events, but not always prioritized.
		Regular meetings and training sessions improved learning. Lower readiness to implement the lesser-known components, such as the facility audit and newsletters.
		Availability and quality of resources
Outer setting	Outside support motivates learning and improvement	Learning and support networks between facilities
		Support from the research team is crucial.
		Alignment with national health priorities and guidelines

### Theme 1 - The E-MOTIVE Intervention Was Well-Packaged for Implementation

*The bundle is based on a combination of well-known and lesser-known interventions.*

Most respondents received the intervention well. They described giving the components of the E-MOTIVE bundle sequentially as per treatment guidelines. All except intravenous tranexamic acid were known to all respondents. A few respondents were familiar with tranexamic acid; however, they reported inconsistent or incorrect use. As one provider quoted:

Some of us knew about Tranexamic acid being used for the management of PPH. We were using it before, but in our own ways. For example, we used to mix 10ml of Tranexamic acid in 500ml of normal saline and run it as an infusion… [FGD #1, Champion doctor]

#### Complexity: Several Strategies are Needed to Make It Work

Most respondents appreciate the interconnected strategies put in place to ensure effective, successful implementation. Healthcare workers appreciated on-site training and praised the intervention packaging for addressing chronic supply shortages. Introducing a well-stocked PPH trolley that was always available, accessible, and movable was seen as a game-changer in PPH care. Some healthcare workers detailed the difficulties they experienced when attending to patients before the intervention began, as one provider narrates…

Before the intervention, we used precious time to mobilize supplies during an emergency……patients progressing to more advanced stages. [FGD #6, nurse-midwife]

Restocking the PPH-trolley was sometimes challenging. Replacement of used supplies is sometimes delayed due to facility pharmacy stock-outs, logistical inconsistencies, or poor communication between shifts of different healthcare workers. Different facilities devised and adapted an accountability mechanism that tasked healthcare workers to restock the trolley at the end of their shift. As one provider narrates:

Participants appreciated the inclusion of local healthcare workers with both clinical and administrative authority, the “E-MOTIVE Champions”. These leaders were perceived as committed and helped facilitate the transition from standard care to the adoption of the E-MOTIVE bundle into routine care. As one respondent narrated.

When a unit lead is also a trainer, and is active in the intervention, it is easier to follow and comply, unlike when the intervention lead is different (not a unit leader) … [FGD #1, Research nurse]

#### Relative Advantage: Tools to Help with Early Detection and Prompt Bundle Initiation

Healthcare workers appreciated and compared diagnostic tools. The calibrated drapes provided a prompt alert that instigated healthcare workers’ prompt support to the patient

We started with non-calibrated drapes that had to be weighed… As we started bundle training, we switched to a calibrated drape with a yellow alert line and a red action line. The lines are more visual, and increase your vigilance for early diagnosis. [FGD #3, Champion doctor]

In addition, Respondents reported that the colour-coded observation checklist helped reinforce regular clinical assessments and documentation.

The checklist was quite useful and detailed. performed, the observed blood loss amount and flow, and the amount and rate of observed blood loss…blood clots or a high flow rate make you more alert. [FGD #2, nurse-midwife]

There were a few challenges with the daily use and organization of basic instruments such as electronic blood pressure machines. Sometimes they were not charged because the chargers were misplaced. As one provider narrates…

The BP machines’ electronic chargers are always in use, healthcare workers use them to charge their phones, and after some time, they move from one place to the next room, easily misplaced… [FGD #2, research nurse]

Respondents perceived that early diagnosis and provision of the bundle reduced severe forms of PPH by arresting excessive bleeding, averting further deterioration of women from severe PPH complications. As one respondent narrated:

We observed a decrease in blood transfusion numbers in the maternity ward. We used to transfuse over 10 units in this ward each month. Blood transfusion is rare now, we think this is because we intervene early…. [FGD #4, Champion doctor]

#### Clarity in Adopting New Practices for PPH Management

Participants expressed that sometimes they lacked clarity when deciding on bundle initiation, specifically whether to initiate a bundle when bleeding is ongoing but not yet 500mls. As one provider described below:

…Sometimes we are uncertain with the calibrated drape. For example, do you trigger a bundle at 450mL… or wait till 500mL? We keep going back and forth discussing what the best way is. [FGD # 3, nurse-midwife]

The respondents engaged in a discourse, and some reiterated the training: to conduct a comprehensive assessment of vital signs, blood flow, and blood loss in the drape before deciding to start the bundle. As one provider recalled:

They (trainers) told us if the calibrated drape points at 300mls, you need to check vital signs: low blood pressure, an increased pulse rate, or a constant trickle of blood, and your clinical judgement. These will help decide on starting the bundle. [FGD # 2, nurse-midwife]

Generally, most respondents felt that being well-equipped reduces their anxiety and frustration. As one provider narrates:

Now we feel at ease. You respond to PPH with confidence. Even when things are not going well, you know you have done your best … [FGD #4, nurse-midwife]

### Individual Characteristics Influencing the Adoption of Intervention into Routine Practices

#### Self-Efficacy: Healthcare Workers Felt that All Components of the Intervention Were within their Scope of Work

Healthcare workers felt empowered and capable of assessing, triggering management, and making decisions, enabling them to deliver a first-response clinical bundle without delay and escalate when necessary. This was seen as a significant step from the usual practice of waiting for a senior person or a doctor to be available. All cadres felt able and skilled to administer intravenous tranexamic acid and do an assessment to determine the cause of bleeding, tasks that were initially left for clinicians.

…examining tears was a physician’s task and not a nurse’s/midwife’s. Now (after the intervention training), it is different. When a nurse calls you for PPH, they have done an assessment and can identify the probable cause of PPH… They trust themselves more. … [FGD #1, Champion doctor]

#### Healthcare Workers’ Willingness to Embrace Practice Change

Respondents still acknowledged challenges in routinely embracing the intervention. Regular clinical assessments were often missed or delegated to the research nurses as most healthcare workers claimed to be too busy. Different facilities handled this differently. As one champion narrates.

We agreed as a team that all postpartum women should be assessed as per the guidelines. It is understandable if one checks vitals twice instead of four times within the hour. We make each other accountable for incomplete charts… [FGD #2, Champion doctor]

### Inner Setting: The Facility’s Setting That Facilitates or Deters Implementation

#### Internal Organization and Structures are Barriers to the Implementation of Clinical Practices

Healthcare workers reported internal processes that may slow adoption and implementation, such as having few healthcare workers per shift and frequent rotations within the facility. Although common, healthcare workers’ rotation destabilizes the maternity workforce, as teams must adapt, relearn, and establish new work dynamics. Some discussants thought that rotating an updated provider is a waste of resources, while others considered rotation necessary for the health facility.

The healthcare workers shortage is beyond the maternity ward and is in every department. Rotating updated staff is usually a setback for the maternity ward. [FGD #2. Champion doctor]

#### Opportunities to Integrate New Interventions Within the Facility Culture

Participants described a well-established knowledge-sharing environment, particularly important during rotation practices or a staff shortage crisis. Healthcare workers from adjacent areas or nursing students are often requested to assist, as it is always beneficial to have extra hands in such emergencies.

… I told her (nursing student) to pull the trolley while I was attending a woman with PPH. We worked together and were able to trigger the bundle in a short time… [FGD # 1, nurse-midwife]

#### Clear Communication is Necessary for Events, but Not Always Prioritized

Respondents reported a lack of clear communication strategies, particularly when handling at-risk women. Often, no clear reports of high-risk women are given to incoming or senior members. As one provider narrated…

Sometimes, we do not report a high-risk woman. We need to work on communication so that we are always prepared. [FGD #2 nurse- midwife]

#### Regular Meetings and Training Sessions Improved Learning

Healthcare workers held regular clinical meetings and practice sessions within the maternity units. Additionally, most facilities initiated weekly meetings to discuss and reflect on severe PPH cases observed. The monthly audit newsletter, produced as part of E-MOTIVE, was a valuable tool in these discussions.

…weekly or monthly meetings are crucial, from which we can discuss all the challenges and brainstorm solutions to them…without the meetings, we may end up going back to business as usual… [FGD #2, Champion midwife]

#### Tension for Change

Most respondents reported their leaders were frustrated with the high burden of PPH and embraced the intervention. Some healthcare workers, however, were skeptical about the new tasks of placing, removing, and weighing a drape. Their views changed over time as they learned and appreciated the added value of drapes. Some facilities were more resistant to change. One research nurse narrates…

We faced resistance in the routine use of drapes. Then, something happened, and maternity ward staff were reshuffled. New staff were more receptive and compliant in using drapes, or they feared being reshuffled, [FGD #2, research nurse]

#### Lower Readiness to Implement the Lesser-Known Components, Such as the Facility Audit and Newsletters

Some respondents were unfamiliar with the monthly facility audit newsletters. Some champions discussed the newsletters with their peers but lacked confidence in sharing with facility administration. Some had positive reports when they reflected on the newsletters, had facility discussions and worked on identified practice gaps. As one respondent narrates…

Our newsletter for June showed five women had severe PPH …. We checked the medical records and reassessed these cases; the majority of women had cervical tears. We discussed how to improve as a team… [FGD #3 Champion midwife]

#### Availability and Quality of Resources

There were occasional supply stock-outs, and relatives had to purchase them. Oxytocin, a key drug for the prevention and management of PPH, was usually available but not kept in ideal conditions. Such inefficiencies result in poor outcomes. As one provider narrated:

Oxytocin is maintained at 2–8°C. Our labour ward doesn’t have a fridge for storage. Every morning, we order it from the main pharmacy and place it in the cooler box with ice packs. Many times, people are busy, and no one follows up to maintain the right temperature. [FGD # 3, Champion doctor]

### Outer Setting

#### Learning and Support Networks Between Facilities

Some respondents shared their successes in alleviating poor PPH outcomes in their facilities. The monthly virtual champions’ meetings were a chance to network and learn from one another. They were positive and flexible, willing to learn from each other and adapt accordingly to achieve success.

… I have heard that facility X has fewer severe cases than us. I would like to learn from them and try to use that to our advantage. [FGD #5, Champion doctor]

#### Support from the Research Team is Crucial

Healthcare workers appreciated the research team’s comprehensive support, which encompassed training, mentoring and discussions of challenging cases.

Having that external support, that external eye that sometimes sees what you do not see in your day-to-day activities, is helpful…can navigate the early challenges together and explore different ways to make things work. The research team has been supportive throughout. [FGD #1, midwife champion]

In addition to the technical support, healthcare workers described being supported in the availability of essential components of the bundle: tranexamic acid, oxytocin, misoprostol, intravenous fluids, and even suture materials and light sources. The trial supported most supplies by providing emergency backup while the facilities continued to procure through their routine processes, which are often challenging.

The study supports us with some supplies and drugs, and beyond, for example, they did a quality check on some samples and advised on what brands of oxytocin and tranexamic acid to procure. They also brought us a fridge to improve our oxytocin storage at the labour ward… [Health worker, FGD#3]

#### Alignment with National Health Priorities and Guidelines

Healthcare workers reported that the intervention was well supported by the local facility administration and regional and district health management teams, as it tackles the most common cause of maternal death. District supervisors participate in facility training and regularly follow their progress. One of the facility teams reported receiving encouragement during a regional supervisory visit.

They (supervisor) came to the delivery unit, asked a lot of questions about the drape and what we do for PPH. She wanted to extend the intervention to other facilities …. *[FGD #4, Healthcare worker]*

Healthcare workers explained that most components of the care bundle were included in the national standard treatment guideline for PPH, except for tranexamic acid, and that the treatment was now given together rather than sequentially.

## Discussion

We explored the early experiences of healthcare workers with a multicomponent strategy to administer a first-response clinical bundle intervention, E-MOTIVE, which included early detection and triggered early management for PPH.^18^

The innovation, E-MOTIVE intervention, was generally well-received; most components were known but not fully implemented, or implemented sequentially. The bundle implementation strategies addressed most chronic challenges, including objective measurement of blood loss, early diagnosis, drug and supply shortages, and provider knowledge, skills, and confidence, all of which affect the implementation of evidence-based guidelines in many LMICs.^33–39^ The implementation strategy targeted both individual and health system factors and empowered healthcare workers with technical skills, self-efficacy, and a supportive environment, including clinical leadership to apply them, which is often not addressed.^16^,^40^ Other literature supports the idea that interventions with multiple components are more effective than single approaches, which have been common in maternal health.^41^

One of the biggest challenges in reducing PPH morbidity and mortality is the inability to make an accurate and early diagnosis. Most LMICs use visual blood loss estimation, which is widely known to be inaccurate.^42^,^43^ The World Health Organization recommends using a quantitative measure of blood loss to achieve objectivity and early detection, especially in the first few hours after birth. Health facilities are also recommended to equip their staff with decision-making skills for prompt, accurate management.^44^

As with any new clinical practice, the calibrated drape introduced new dilemmas, particularly in decision-making around action triggers, disposal, and sustainability, which were often iterated within the facilities. Such new experiences were shared, helping to understand and further improve the implementation process.

Facility audits and feedback mechanisms are known to be useful quality measures in maternal health; however, they are not always understood or followed due to a lack of knowledge or time, poor communication, and fear of punitive feedback.^45–47^ This finding underscores the urgent need to improve data literacy among healthcare workers caring for patients.^48^ Such audits and other data sources can be harnessed for quality improvement in different service provision beyond the study. While there is a global effort for standardizing data use.^49^ These efforts are often fragmented in LMICs.

Clinical leadership skills were key in ensuring adherence to the intervention.^50^ The local clinical leads, the E-MOTIVE champions, were well-engaged, motivated, and accountable This highlights the importance of having the right individuals in these clinical leadership roles, who serve as role models and change-makers. Several studies in sub-Saharan African countries have reported such clinical leads to be key in disseminating and uptake of clinical guidelines and practices.^22^,^50–52^

Our results underscore an important inner settings domain that is crucial to implementation. Some organizational challenges in one facility became a reinforcement in another. For example, while staff rotation was predominantly viewed as a barrier due to the loss of trained personnel, in one instance, it inadvertently facilitated adoption by introducing more receptive staff to the unit, a finding that highlights the unpredictable nature of organizational change. Such findings show that even disliked changes can have unexpected and positive results.

### Methodological Consideration

This study was conducted three months after the initiation of the E-MOTIVE intervention, during which we believe the teams were still learning and improving their skills and processes. We included all intervention implementers, such as local healthcare workers, champions, and research nurses, who we believe had firsthand experience. Since most facilitators were known to the healthcare workers, there was a risk that respondents would report only positive experiences; however, this did not occur, as some expressed challenges they faced and needed clarification. The FGD discussions were conducted after the research team visited the facility and reviewed work processes in the maternity ward. The last FGD, conducted with all champions, provided a valuable opportunity to understand both collective and facility-specific challenges, as well as how the champions’ peer support network could facilitate the transfer of effective practices across settings.

### Implications for Practice

Most interventions have been unsuccessful in reducing PPH because they have addressed skills, prevention, or supply shortages in silos. The E-MOTIVE intervention is promising, as it tackles chronic challenges by targeting facility preparedness and healthcare workers’ skills and empowerment. The success of this innovation can be attributed to the extensive formative work undertaken prior to implementation. The formative work included codesign workshops that shaped the intervention and its delivery strategies.^13^,^16^ Such contextualization and stakeholder inclusion during the planning phase of an intervention are ideal for ensuring that local challenges are effectively addressed.

The intervention empowered healthcare workers through on-site training, clinical leadership, and, during frequent meetings, a space for brainstorming challenges and developing local solutions. This created strong local leadership and ownership within facilities and should be further explored.

Use of calibrated drapes was novel in the study; these drapes were procured externally and are not available in the country. As with many new tools, there was an initial resistance due to misconceptions about extra work. Stakeholders need to explore local innovations that can objectively measure blood loss, be introduced in facilities, and be supported for consistent use. It is yet to be determined how the intervention can be adopted in places without drapes that still rely on routine measures of blood loss.

There is an underexploited opportunity in using facility audits to improve the quality of healthcare services during childbirth and empower facilities to use their locally generated data to make meaningful changes.^45–47^ Furthermore, the intervention introduced several new tools, including an enhanced postpartum monitoring checklist that incorporates uterine tone and bleeding description, which can be scaled up to improve monitoring.

Furthermore, several challenges need to be addressed, including the local organizational culture of rotating staff from the maternity wards to other areas within or outside facilities, and improving communication strategies within the maternity wards and the facilities’ administrations.

Several other factors affecting implementation, such as a strong political commitment and continuous maternal death audits, led facilities to want to improve their clinical practices and outcomes.^53^ As PPH is a leading cause of poor outcomes, it made sense to work to reduce it within the study setting.

### Strengths and Limitations

We report on the early implementation experiences from one LMIC country, Tanzania, as a learning point and an opportunity to address implementation challenges, especially for countries, programs or health facilities that plan to introduce or scale up E-MOTIVE in their local context. We had first-hand information on the challenges and opportunities, and how the different components fed into each other for the success. We also had opportunities to address the dilemmas and lack of clarity in some areas, and to offer support to the implementing teams.

Most of the respondents were already familiar with some of the research teams, which could have led to social desirability bias during discussions. We also used natural FGD discussions, which could be influenced by established power dynamics and FGD think. However, we observed candid discussions on relevant issues. The natural FGDs brought in rich contextual and implementation data and were efficient. The champion FGD built upon most of what was discussed earlier, providing a good learning curve. The CFIR framework was used for analysis only, not for planning or data collection; however, its components fit well with the framework. It was challenging to distinguish between the innovation and the process, as the intervention encompasses both the bundle and its strategies; therefore, we presented and discussed them together.

Findings from this study are reported within the context of the main trial, which had implementation support to ensure the smooth integration of the intervention into routine care. Research nurses often participated in clinical care due to the severe shortages of healthcare workers in study facilities. The main trial supported the initial availability of drugs, medical supplies, equipment and trolleys. The replacement and ensuring that all supplies were available were later led by local champions. Because this exploratory qualitative study mainly focused on the early implementation experiences of facility-based teams, broader outer setting factors—such as supply chain systems, referral constraints, and alignment with national obstetric guidelines—were not examined in detail. Future research could look into these wider health system influences to better understand their role in sustaining and scaling the intervention.

## Conclusion

This study demonstrates that from the perspective of frontline healthcare workers in Tanzania, the E-MOTIVE bundle is not only acceptable but empowering, addressing chronic systemic challenges while building local capacity and confidence. The bundle included detection and management components that needed to be completed in a timely manner, and the accompanying implementation strategies, such as PPH-trolley and champions, were well received. Several areas required additional support, including clarifying trigger points and maximizing the use of audit newsletters in routine quality improvement. The study emphasizes the advantages of using interconnected strategies that address known challenges. There are several opportunities for success, such as tapping into healthcare workers’ potentials, local clinical leadership, inter-facility networking and peer-to-peer support. As countries plan to scale up the E-MOTIVE bundle, they must invest not only in the clinical components but also in strengthening the “software” of implementation, specifically, in supporting local clinical leaders, building data literacy for quality improvement, and developing health system strategies to mitigate the disruptive effects of staff turnover. These investments are essential to translate the promise of the E-MOTIVE trial into sustained impact on maternal survival.

## Data Availability

The data used to analyze during this manuscript is available from the corresponding author on reasonable request.

## Abbreviations

AMSTL, Active Management of the Third Stage of Labour; CFIR, Consolidated Framework for Implementation Research; E-MOTIVE, Early detection, uterine Massage, Oxytocic, Tranexamic acid, Intravenous fluids and escalate; FGD, Focus Group Discussion; LMIC, Low- and middle-income countries; PPH, Postpartum Haemorrhage; WHO World Health Organization.

## Patient and Public Involvement

Patients and/or the public were not involved in the design, or conduct, or reporting, or dissemination plans of this research.
